# Water Deficit Affects Primary Metabolism Differently in Two *Lolium multiflorum*/*Festuca arundinacea* Introgression Forms with a Distinct Capacity for Photosynthesis and Membrane Regeneration

**DOI:** 10.3389/fpls.2016.01063

**Published:** 2016-07-25

**Authors:** Dawid Perlikowski, Mariusz Czyżniejewski, Łukasz Marczak, Adam Augustyniak, Arkadiusz Kosmala

**Affiliations:** ^1^Institute of Plant Genetics, Polish Academy of SciencePoznań, Poland; ^2^Institute of Bioorganic Chemistry, Polish Academy of SciencesPoznań, Poland

**Keywords:** chloroplast aldolase, drought, forage grasses, membrane regeneration, photosynthetic activity, primary metabolites

## Abstract

Understanding how plants respond to drought at different levels of cell metabolism is an important aspect of research on the mechanisms involved in stress tolerance. Furthermore, a dissection of drought tolerance into its crucial components by the use of plant introgression forms facilitates to analyze this trait more deeply. The important components of plant drought tolerance are the capacity for photosynthesis under drought conditions, and the ability of cellular membrane regeneration after stress cessation. Two closely related introgression forms of *Lolium multiflorum*/*Festuca arundinacea*, differing in the level of photosynthetic capacity during stress, and in the ability to regenerate their cellular membranes after stress cessation, were used as forage grass models in a primary metabolome profiling and in an evaluation of chloroplast 1,6-bisphosphate aldolase accumulation level and activity, during 11 days of water deficit, followed by 10 days of rehydration. It was revealed here that the introgression form, characterized by the ability to regenerate membranes after rehydration, contained higher amounts of proline, melibiose, galactaric acid, *myo*-inositol and *myo*-inositol-1-phosphate involved in osmoprotection and stress signaling under drought. Moreover, during the rehydration period, this form also maintained elevated accumulation levels of most the primary metabolites, analyzed here. The other introgression form, characterized by the higher capacity for photosynthesis, revealed a higher accumulation level and activity of chloroplast aldolase under drought conditions, and higher accumulation levels of most photosynthetic products during control and drought periods. The potential impact of the observed metabolic alterations on cellular membrane recovery after stress cessation, and on a photosynthetic capacity under drought conditions in grasses, are discussed.

## Introduction

The sedentary life style of plants exposes them to many unfavorable environmental conditions, limiting their growth and development. Abiotic stresses, such as drought (water deficit), salinity, flooding, and low temperature strongly affect plants during their life cycle. Among those factors water deficit is one of the most important, disturbing plant metabolism, inhibiting their growth and reducing productivity worldwide (Bray et al., 2000; Zhang et al., 2005). During their evolution plants developed many strategies for surviving water deficit, namely: drought avoidance, drought tolerance, drought escape, and recovery after drought cessation (Fang and Xiong, 2015). Numerous anatomical, physiological and molecular components of plant performance, including root parameters, leaf features, osmoprotection system, ROS scavenging system, membrane stability and photosynthetic capacity, influence a plant’s response to drought (Fang and Xiong, 2015).

Plant productivity depends mainly on photosynthesis, which is one of the first and most sensitive physiological processes affected by drought (Lawlor, 2002; Munns, 2002). Several studies have been conducted in order to improve our knowledge about drought-induced inhibition of photosynthesis (Cornic, 2000; Lawlor, 2002; Chaves et al., 2003; Flexas et al., 2004). These studies have shown that the inhibitory effects of drought on photosynthesis could be associated with low CO_2_ availability, due to limitations of its diffusion through the stomata (stomatal limitations) and/or due to non-stomatal limitations, including both diffusive (reduced mesophyll conductance) and metabolic (photochemical and enzymatic limitations) processes (Lawlor, 2002; Lawlor and Cornic, 2002; Flexas et al., 2004). Nevertheless, there is an ongoing debate on whether drought stress influences photosynthesis more by stomatal or non-stomatal alterations (Flexas and Medrano, 2002). Stomatal closure has been identified as an early response to decreasing soil water potential, or to a decline in leaf turgor due to fall in a relative water content, and as an efficient way to reduce water loss in drying field conditions. Simultaneously, a decreasing stomatal conductance limits carbon uptake into leaves, which affects the photosynthesis during mild to moderate drought (Cornic, 2000; Lawlor, 2002). However, during prolonged and more severe periods of drought metabolic limitations, including a reduction of crucial photosynthetic enzymes’ activities, may also significantly reduce CO_2_ assimilation (Flexas et al., 2004; Signarbieux and Feller, 2011). It was suggested that these limitations are mainly associated with enzymes of the Calvin cycle (Chaves et al., 2003). This relationship was demonstrated for the inhibition of ribulose-1,5-bisphosphatecarboxylase (Rubisco; Chaves et al., 2003), decreases in a total Rubisco activity and protein content (Flexas et al., 2006), and an inhibition of Rubisco activase (Lawlor, 2002). Furthermore, the rate of photosynthesis could be limited not only by a carboxylation of Rubisco but also by a ribulose-1,5-bisphosphate regeneration capacity, which could be reduced mainly by a decreased accumulation of plastid fructose-1,6-bisphosphatase (Kossmann et al., 1995), an inhibition of sedoheptulose-1,7-bisphosphatase (Harrison et al., 1998) and a reduced accumulation of chloroplast fructose-1,6-bisphosphate aldolase (pFBA; Haake et al., 1998, 1999; Uematsu et al., 2012). As it was demonstrated earlier, alterations in photosynthetic carbon metabolism in response to drought could also be strongly associated with accumulation levels of several classes of primary metabolites, mostly plant hormones, osmoprotectants and ROS scavenging particles which are crucial to develop drought tolerance (Chandler and Robertson, 1994). Osmoprotectants are important soluble metabolites and belong to amino acids and carbohydrates, sharing common characteristics, such as small molecular weight and non-toxic character, thus they can be accumulated in large quantities without being harmful for the cell functioning (Rontein et al., 2002). Under osmotic stress plants produce organic osmolytes from a group of soluble sugars, such as fructose or sucrose, organic alcohols, such as *myo*-inositol, complex sugars, such as trehalose or fructans or amino acids, such as proline and modified amino acids, such as glycine betaine (Kido et al., 2013). These compounds could also function as chaperone-like molecules stabilizing membranes and maintaining the activity and stability of the enzymes crucial for a proper functioning of cell metabolism (Xoconostle-Cazares et al., 2010).

*Festuca arundinacea* (tall fescue) is one of the most drought tolerant grass species in the *Lolium-Festuca* complex. *Lolium multiflorum* (Italian ryegrass) has high yielding capacity but significantly lower levels of tolerance to environmental stresses, such as drought, compared to *F. arundinacea*. *L. multiflorum* and *F. arundinacea* hybridization enables the assembly of complementary characters of both species within a single genotype (Kosmala et al., 2012; Perlikowski et al., 2014). The *L. multiflorum/F. arundinacea* introgression forms were shown in our earlier work to be excellent plant materials for dissecting drought tolerance of *F. arundinacea* into several crucial components (Perlikowski et al., 2014). Two introgression forms (4/10 and 7/6) were selected for further research performed at different levels to go deeper into the molecular mechanisms of drought tolerance existing in the group of *Lolium-Festuca* forage grasses. The introgression form 4/10 with better yield performance under simulated drought conditions in the field (14 weeks), and a faster re-growth after stress cessation, was also characterized by stronger membrane regeneration during recovery after 11 days of drought in simulated pot conditions. It was manifested by the electrolyte leakage parameter, describing the level of membrane stability. This parameter increased significantly on the 11th day of drought application in the two introgression forms, although after re-watering it returned to the values calculated for the conditions before drought initiation only in the form 4/10. On the other hand, the form 7/6 was characterized by a lower yield potential after 14 weeks of drought in the field, and a lower ability of re-growth after rehydration. However, this form was also shown to possess a greater level of photosynthesis capacity during 11 days of drought treatment in pot conditions, compared to the form 4/10, as manifested by CO_2_ assimilation level [μmol(CO_2_) m^-1^s^-1^] (values marked below with the same letter did not differ statistically at *P* = 0.05, according to Tukey HSD test). This level was significantly higher in the form 7/6 on the 11th day of drought treatment (7.42 ± 0.16*b*), compared to the genotype 4/10 (6.58 ± 0.17*c*; Perlikowski et al., 2014). Our earlier work (Perlikowski et al., 2014) demonstrated that more efficient photosynthesis during drought in the form 7/6 was, with a high probability, not associated with the photoactivity performance, since no differences between the analyzed introgression forms in the level of chlorophyll fluorescence parameters were observed under water deficit. Furthermore, it was also shown in our previous work that under drought conditions CO_2_ assimilation rate was not limited by stomatal aperture; both introgression forms significantly reduced stomatal aperture under drought, compared to the control conditions but with a closely similar level of stomatal conductance the form 7/6 revealed a significantly higher level of CO_2_ assimilation rate, compared to the form 4/10. We suggested that this greater capacity of photosynthesis could be due to a higher efficiency of the Calvin cycle in that introgression form. After pre-screening of protein profiles based on 2-D maps, it was found that the accumulation level of pFBA (EC 4.1.2.13) was higher in the form 7/6 (Perlikowski et al., 2014). These two closely related *L. multiflorum/F. arundinacea* introgression forms, 4/10 and 7/6, were applied in the research presented herein.

In this study, we hypothesize that (i) the introgression form 7/6, with more intensive CO_2_ assimilation level during drought, will be characterized by higher pFBA accumulation and activity levels, which could be a crucial component of non-stomatal machinery involved in a regulation of photosynthetic efficiency in the *Lolium-Festuca* forage grasses; (ii) this phenomenon will also be accompanied by a higher accumulation level of primary photosynthetic metabolites in this form. Moreover, we hypothesize that (iii) a stronger regeneration capacity of the introgression form 4/10, including a membrane regeneration process after stress cessation, could be associated with higher accumulation levels of key metabolites, including osmoprotectants responsible for a protection of crucial proteins, and other important cell components. Thus, the research presented herein performed on the two introgression forms -4/10 and 7/6 involved: (i) Western blot experiments to confirm pFBA accumulation level during water deficit and recovery periods, accompanied by pFBA activity measurements and (ii) a primary metabolite profiling under stress conditions and after stress cessation, using GC - MS.

## Materials and Methods

### Plant Materials

Plant materials used in the present research involved two *L. multiflorum/F. arundinacea* introgression forms (genotypes 4/10 and 7/6) obtained after four rounds of backcrossing of *L. multiflorum* (4x) × *F. arundinacea* (6x) hybrid to *L. multiflorum* (4x). These plants were selected earlier from a larger population in the field conditions with respect to their drought tolerance, as described by Perlikowski et al. (2014). After this selection, the two forms, each one in four biological replicates, were transferred to pots (1.75 dm^3^), containing a sand:peat (1:3) mixture. The experiment of 11 days of water deficit, followed by 10 days of re-watering was performed in a growth chamber at a temperature of 22/17°C (16 h day/8 h night, light of 400 μmol(quanta) m^-2^ s^-1^, HPS “Agro” lamps, Philips, Brussels, Belgium), 30% relative air humidity and watering completed. The level of soil water content decreased from 63% of field water capacity observed in control conditions down to approximately 3% on the 11th day of stress duration. After 10 days of re-watering this capacity increased to the value observed before drought treatment (Perlikowski et al., 2014). The physiological measurements summarized in the introduction, were performed during this experiment. The leaf tissue sample (100 mg) was collected before drought treatment (control), at three different time-points of drought (after 3, 6, and 11 days of drought), and after 10 days of subsequent re-watering, every time from each replicate, and frozen in liquid nitrogen.

### Identification of Aldolase cDNA Sequences

Full length cDNA sequences encoding chloroplast 1,6-bisphosphate aldolase (pFBA) were obtained by RACE reaction using commercial kit (5′/3′ RACE Kit, 2nd Generation - ROCHE^®^). Initial primers (forward primer – TTCGAGGAGACCCTCTACCA; reverse primer – GGCTACAGTGCCCTCTCAAG) were designed on the basis of pFBA mRNA sequence of *Brachypodium distachyon* available in NCBI database [gi| 357157398|ref|XM_003577737.1|]. Special primers for RACE reaction were designed on the basis of sequenced initial fragment of pFBA cDNA:

(1) SP-F1 - GACTGTAGATGGCAAGAAGATTGTTGAC
(1) SP-F2 - CCAATTGTTGAGCCTGAGATCATG
(1) SP-R1 - CTACTAGCACTCTCTCCATAGGTAGATA
(1) SP-R2 - ATCAGTAGCTGTAGTTCTTGACGAACAT
(1) SP-R3 – TTCTCTGGAGGAGCTTGAGAGTGTA.

The PCR and RACE products were purified by QIAEXII Gel Extraction Kit (Qiagen), and ligated into the pGEM-T Easy vector (Promega). Vectors containing the ligated product were transformed into *Escherichia coli* strain XL1 Blue, and multiplied plasmids from clones selected with X-Gal and IPTG were extracted using QIAprep Spin Miniprep Kit (Qiagen). The obtained plasmids were sequenced (Molecular Biology Techniques Laboratory, Faculty of Biology, Adam Mickiewicz University, Poznań) using SP6 and T7 primers. The obtained cDNA sequences were aligned with BioEdit software (ver 7.2.5).

### Analysis of Aldolase Accumulation Level

To estimate a pFBA protein accumulation level a Western blot analysis was performed, with the antibody directed against pFBA. The antibody was produced by Agrisera^®^ company^1^ using a rabbit host immunized with a highly specific pFBA 15 amino acid peptide (TFEVAQKVWAETFYY). The peptide was selected on the basis of comparison between pFBA sequence of the two analyzed introgression forms, and available in database sequence of a cytosolic enzyme of *B. distachyon* (XM_003564823.1) to avoid a cross-reaction of the anti-pFBA antibody with a cytosolic FBA. The detailed protocols for a protein extraction and Western blotting were as described by Pawłowicz et al. (2012). Briefly, 10 μg of chloroplast proteins from each time-point and introgression form in three biological replicates and standard samples were separated by 12% SDS-polyacrylamide gel electrophoresis and electroblotted onto nitrocellulose membranes (Bio-Rad). Immunodetection was performed with a rabbit polyclonal antibody (diluted 1:4000; Agrisera). The antigen–antibody complexes were detected using a chemiluminescent detection system with a secondary anti-rabbit IgG–horseradish peroxidase conjugate (diluted 1:20 000; Sigma) and a chemiluminescent substrate (Westar Supernova – Cyanogen) and the products intensities were estimated using ImageJ software.

### Analysis of Aldolase Activity

The pFBA activity was measured according to a modified Sibley-Lehninger method (Sibley and Lehninger, 1949; Willard and Gibbs, 1968). A protein extract from chloroplasts was prepared according to a modified method used by Kosmala et al. (2012). Briefly, 1g of frozen leaf material in three replicates for each sample, was ground in a liquid nitrogen, suspended in 4 ml of chloroplast isolation buffer (Sigma–Aldrich), shaken, filtered through a mesh 100 nylon (Sigma–Aldrich) and centrifuging 3 min at 200 *g* at 5°C. The collected supernatant was subsequently centrifuged for 15 min at 900 *g* at 5°C, and the washed chloroplast pellet was suspended in 2 ml 0.1 M phosphate buffer (0.1 M Na_2_HPO_4_) with 3% Triton X100 and shaken 5 min in 1000 rpm. The collected supernatant was used to determine the pFBA activity. To 2 ml tubes 50 μl of 0.06 M fructose-1,6-bisphosphate and 140 μl of incubation buffer (0.05 M 2,4,6 trimethylpyridine, 0.08 M hydrazine sulfate, 0.3 mM sodium iodoacetate) pH 7.4 were added and pre-incubated in water bath during 3 min at 30°C. The volume of 100 μl of chloroplast extract was added and incubated at 30°C for 2 h. After the incubation, tubes were chilled and 300 μl of 10% trichloroacetic acid was added to stop the reaction. For each sample one tube was treated as a reagent blank and was filled with 300 μl of 10% trichloroacetic acid before proceeding. After centrifugation at 10000 *g*, 100 μl of collected supernatant was pre-incubated with 100 μl of 0.75 M NaOH at room temperature for 10 min and then incubated at 30°C water bath for 10 min with addition of 100 μl of 0.1% 2,4-dinitrophenylhydrasine. After that step, samples were mixed with 700 μl of 0.75 M NaOH and the absorbance measurements were performed with reference to a reagent blank using a spectrophotometer with 540 nm wavelength. A standard curve was prepared as follows: 2 ml tubes in two replicates were filled in order with 25, 50, 75, and 100 μl of 0.01 mM D-glyceraldehyde and filled up with water to a final volume of 100 μl. In the next step, 100 μl of 2,4-dinitrophenylhydrasine solution was added and samples were incubated at 30°C water bath for 10 min. After incubation, 800 μl of NaOH was added and after 3 min of incubation the absorbance was measured with 540 nm with reference to a blank sample (100 μl of water plus reagents). The amount of produced trioses in the pFBA assay was read according to a standard curve and presented after calculation as μg of glyceraldehyde produced by 1 g of plant sample during 1 h.

### Metabolite Profiling

Analysis of primary metabolites accumulation was performed with slight modifications according to the protocol described earlier by Wojakowska et al. (2015). This protocol is presented briefly in the following sub-sections.

#### Materials and Reagents

Solvents used for extraction and GC-MS analyses were MS grade methanol, methylene chloride, isopropanol, ribitol; derivatization reagents for GC-MS analyses were - MSTFA, *O*-methylhydroxylamine hydrochloride, pyridine and alkanes (C10–C36) used as retention index standards purchased from Sigma–Aldrich (Poznań, Poland). A deionized water was purified by Milli-Q system Direct Q3 (Millipore, Bedford, MA, USA). A homogenization was performed with the MM400 (Retsch GmbH, Haan, Germany) homogenizer. Centrifugation was done with the EBA21 centrifuge (Hettich, Tuttlingen, Germany).

#### Extraction of Metabolites

The amount of 100 mg of dried, powdered leaf material sample was transferred to 2 ml plastic tubes with two stainless steel balls, and 1.5 ml of 80% methanol in deionized water was added. Ribitol (25 μl of 1 mg/ml solution) was added to each sample as the internal standard. The samples were homogenized for 10 min at 1800 rpm, sonicated for 15 min and centrifuged for 15 min at 12000 rpm, followed by filtering through PTFE syringe filters 0.45 μm GHP ACRODISC 1 (Waters, Milford, CT, USA). The volume of 300 μl of each sample was transferred to a new tube and evaporated in a SpeedVac concentrator. A dried extract was then derivatized with 50 μl of methoxyamine hydrochloride in pyridine (20 mg/ml) at 37°C for 90 min with agitation. The second step of derivatization was performed by adding 80 μl of MSTFA and an incubation at 37°C for 30 min with agitation. Samples were subjected to GC/MS analysis directly after a derivatization. Each sample was prepared in four replicates. The compounds were considered “identified” when they met the identification criteria established by the GC software used (LECO ChromaTOF), namely: identity score higher than 700, Mass Threshold higher than 10 and a matched retention index.

#### GC/MS Analysis

The analysis for separation was performed using the Agilent 7890A gas chromatograph (Agilent Technologies) connected to Pegasus 4D GCxGC-TOFMS mass spectrometer (Leco). A DB-5 bonded-phase fused-silica capillary column of 30 m length, 0.25 mm inner diameter and 0.25 μm film thickness (J&W Scientific Co., USA). The GC oven temperature program was as follows: 2 min at 70°C, raised by 8°C/min to 300°C and held for 16 min at 300°C. The total time of GC analysis was 46.75 min. Helium was used as the carrier gas at a flow rate of 1ml/min. The volume of 1 μl of each derivatized sample was injected in a splitless mode. The initial PTV (Programmed Temperature Vaporization) injector temperature was 20°C for 0.1 min and then raised by 600°C/min to 350°C. The septum purge flow rate was 3 ml/min and the purge was turned on after 60 s. The transfer line and ion source temperatures were set to 250°C. In-source fragmentation was performed with 70 eV energy. Mass spectra were recorded in the mass range 35–650 m/z.

### Analysis of Mass Spectra

Data acquisition, automatic peak detection, mass spectrum deconvolution, retention index calculation and library search were done by Leco ChromaTOF-GC software (v4.51.6.0). To eliminate retention time shift and to determine the retention indexes (RI) for each compound, the alkane series mixture (C–10 to C–36) was injected into the GC/MS system. The metabolites were automatically identified by library search (Replib, Mainlib, Fiehn library) with a similarity index above 700 and retention index ±10. All known artifact peaks including alkanes, plasticizers, column bleed, MSTFA artifact and reagent peaks were not considered in the final results. To obtain accurate peak areas for the deconvoluted components, unique quantification masses for each component were specified and the samples were reprocessed. The obtained metabolite data was normalized relatively to the quant mass peak of internal standard (Ribitol – 217) in each sample before statistical analysis.

### Statistical Analysis

The normalized mass spectral intensity Log-transformed data (base 2) was subjected to statistical analysis. Two-way analysis of variance (ANOVA) with a genotype and treatment as classification factors, Fisher’s least significant difference (LSD) and PCA was made using STATISTICA 10 software (StatSoft, Tulsa, OK, USA). The PCA was carried out by eigenvalue decomposition of data correlation matrix. The significant effects of genotype, time and genotype × time interaction were selected using the family wise error rate less than 1%. Fisher’s LSD of samples at 1% was used. Heatmaps for a difference between means of time-points and the control were prepared.

## Results

### The Accumulation Level and Activity of Chloroplast Fructose Bisphosphate Aldolase during Drought and Rehydration

The RACE analysis performed on total RNA extracted from the two introgression forms allowed the identification of two pFBA mRNA sequences in each form (**Supplementary Figure S1**). The overall length of the identified sequences with 3′ and 5′ UTR varied from 1423 to 1430 nucleotides, whereas the length of the coding region was the same for each identified sequence and covered 1164 nucleotides. This coding region showed 99% of similarity to *B. distachyon* sequence used earlier for primers designed to clone pFBA from the analyzed introgression forms (**Supplementary Figure S1**). The identified sequences were characterized by several single nucleotide polymorphisms within a coding region, and modifications within 3′ and 5′ UTR (**Supplementary Figure S1**). The predicted protein sequence for the analyzed mRNAs covered 388 amino acids, including 37 amino acids of the chloroplastic transit sequence (**Supplementary Figure S2**). The predicted molecular mass of the protein was 42.05 and 38.48 kDa with and without a transit sequence, respectively. Its predicted isoelectric point was 5.51, and between the different predicted here protein sequences three amino acid modifications were found (**Supplementary Figure S2**).

**FIGURE 1:**
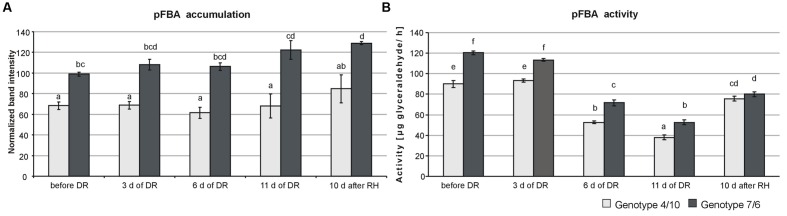
**The activity and accumulation levels of chloroplast fructose bisphosphate aldolase (pFBA) at five time-points: before drought, after 3, 6 and 11 days of drought (DR), and 10 days of re-watering (RH) in *Lolium multiflorum/Festuca arundinacea* introgression forms.** The bars represent a calculated mean value of enzyme activity expressed in μg glyceraldehyde produced by pFBA during 1 h of incubation at 30°C **(A)** and a mean of pFBA bands intensities values **(B)**. Error bars represent standard deviations. The letters indicate groups of means that do not differ significantly at a significance level of 0.01 (Fisher’s LSD-test).

The proteomic assays revealed a significantly higher accumulation level (**Figure 1A**) and total activity (**Figure 1B**) of pFBA in the 7/6 introgression form at all the time-points of drought treatment, compared to the 4/10 form. However, in both forms a significant decrease of pFBA activity after 6 and 11 days of drought was observed, compared to the control conditions and initial days of stress duration. On the other hand, after 10 days of rehydration an increase of enzyme activity was revealed but without significant differences between the two forms (**Figure 1B**).

### The Accumulation Level of Primary Metabolites during Drought and Rehydration

#### Metabolite Accumulation Dynamics

**FIGURE 2:**
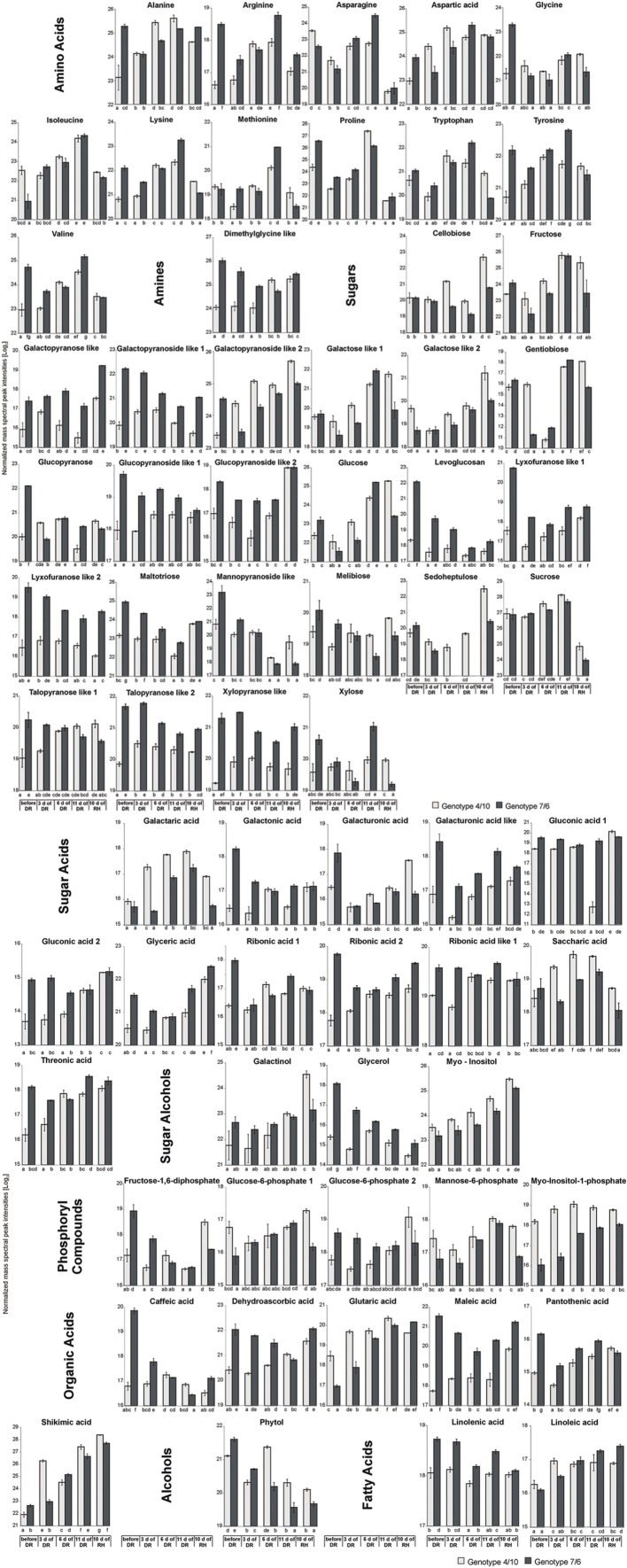
**The accumulation levels of analyzed metabolites at five time-points: before drought, after 3, 6, and 11 days of DR, and 10 days of RH in *L. multiflorum/F. arundinacea* introgression forms.** The bars represent a mean value (over replications) for Log_2_ transformed mass spectra peak intensities. Error bars represent none weighted standard errors. The letters indicate groups of means that do not differ significantly at a significance level of 0.01 (Fisher’s LSD-test).

**FIGURE 3:**
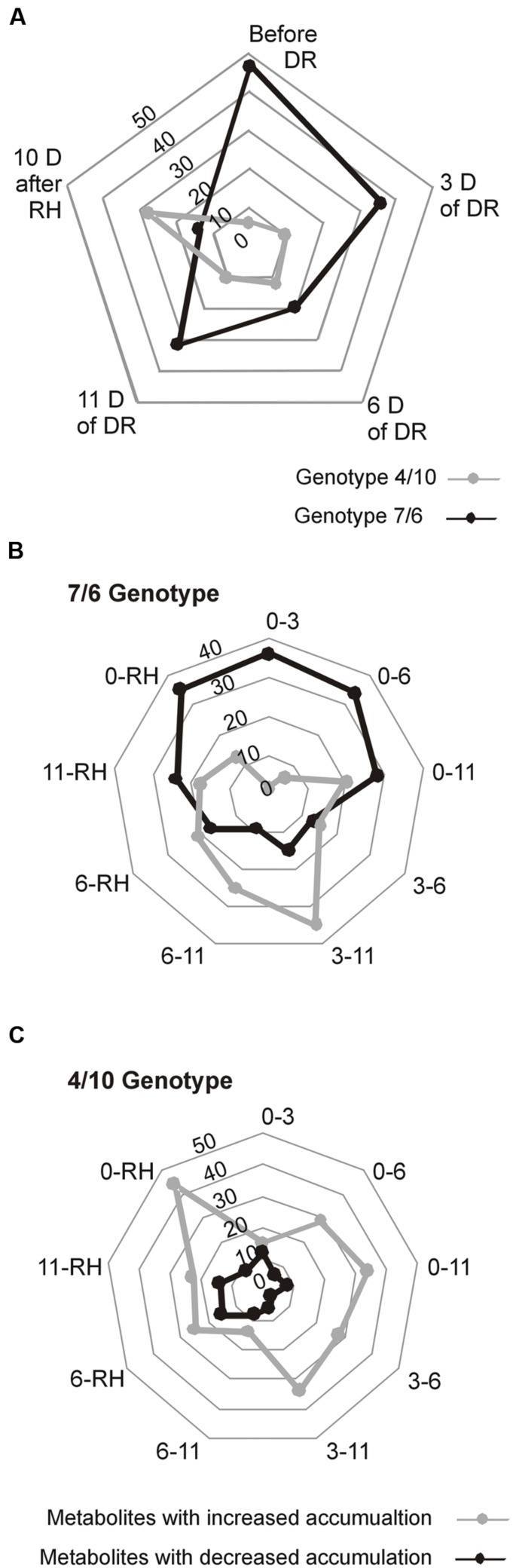
**The overall statistical comparison of differences in accumulation levels of the analyzed metabolites at five time-points: before drought, after 3, 6, and 11 days of DR, and 10 days of RH in *L. multiflorum/F. arundinacea* introgression forms.** **(A)** Numbers of metabolites with a significantly higher accumulation level between the analyzed forms at the particular time-points. **(B)** Numbers of metabolites with a significant increase or decrease in an accumulation level between time-points in the 7/6 form. **(C)** Numbers of metabolites with a significant increase or decrease in an accumulation level between time-points in the 4/10 form.

A total of 937 different metabolite compounds were identified, and 66 were selected for further analysis. These 66 were present in all the biological replicates, and had a similarity index value above 700 and/or were manually identified using a comparison of their retention time with Golm metabolite VAR5 library data^2^, and after one-way ANOVA pre-selection had *p*-value lower than 0.01 (**Supplementary Table S1**). The analyzed metabolites were further divided into nine classes, including amino acids, amines, sugars, sugar acids, sugar alcohols, phosphoryl compounds, organic acids, alcohols, and fatty acids (**Figure 2**). The further statistical analysis revealed that 50 compounds presented significant genotype dependent differences, 63 time-point dependent differences and 60 compounds revealed a significant interaction between a genotype and a time-point (**Supplementary Table S1**). The introgression form 7/6 was characterized by a significantly higher accumulation level of primary metabolites, including 47 in the control conditions and 32 after 11 days of drought, compared to the 4/10 form. This latter plant expressed a higher accumulation level of six metabolites in the control conditions and 10 after 11 days of drought, compared to the form 7/6. Only after rehydration did the form 4/10 reveal 28 metabolites with a significantly higher accumulation level than the form 7/6. At this time-point the form 7/6 expressed only 14 metabolites with higher accumulation levels than the form 4/10 (**Figure 3A**).

**FIGURE 4:**
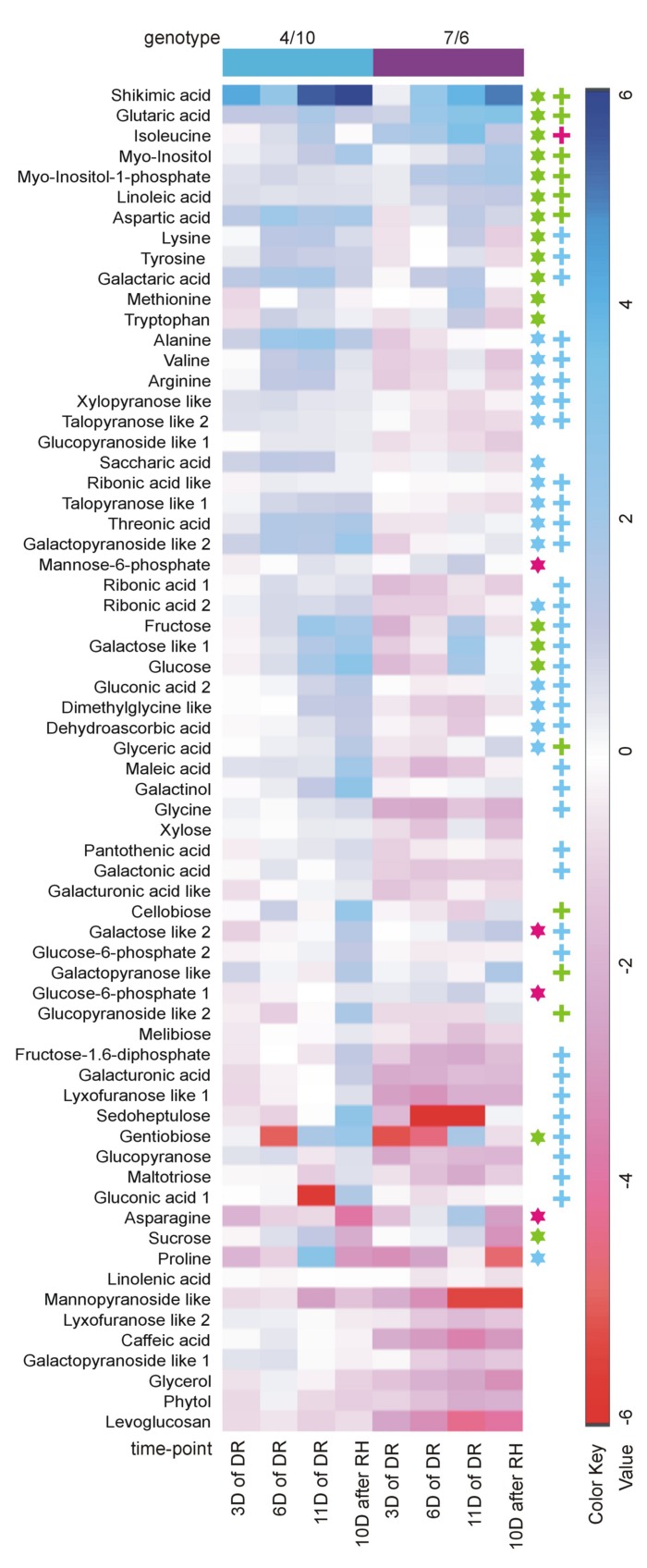
**The accumulation levels of the analyzed metabolites (relative to control values, calculated for mean Log_2_ transformed mass spectral peak intensities) at four time-points of the experiment: after 3, 6, and 11 days (D) of DR, and after 10 days of RH in the 4/10 and 7/6 *L. multiflorum/F. arundinacea* introgression forms.** The values lower than the control are shown in shades of red, and the values higher than the control are shown in shades of blue. The asterisks indicate metabolites with a significant increase of an accumulation level after 11 days of drought: blue in the 4/10 form, magenta in the 7/6 form and green in both introgression forms. The crosses indicate metabolites with a significantly higher accumulation level after 10 days of re-watering: blue in the 4/10 form, magenta in the 7/6 form and green in both introgression forms.

**FIGURE 5:**
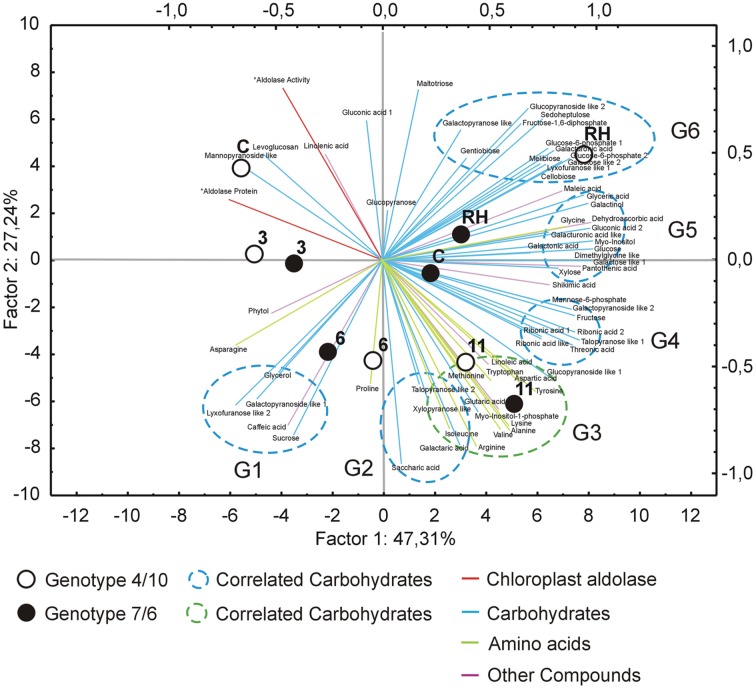
**The PCA of the analyzed metabolites accumulation level using the first two principal components with the highest variance.** Close circles represent the 7/6 *L. multiflorum/F. arundinacea* introgression form, open circles – the 4/10 form. The time points are labeled, as follows: C, control; 3, the 3rd day of drought; 6, the 6th day of drought; 11, the 11th day of drought; RH, 10 days after re-hydration. Blue lines represent carbohydrate metabolites, green – amino acids, purple - other metabolites. Dotted line circles represent correlation groups (G1-6).

The analysis of changes in accumulation levels between the time-points in these two introgression forms revealed that during drought duration the patterns of metabolite dynamics were different in both plants. Between the control conditions and drought time-points more metabolites were significantly down-regulated in the 7/6 form, especially at the beginning of drought treatment between – the control and the 3rd day of stress period, with 36 metabolites decreasing their accumulation levels. The abundance of these metabolites started to be up-regulated between the 3rd and 11th day of drought, with 35 metabolites increasing their abundance (**Figures 3B** and **4**). Different patterns of metabolite dynamics were observed in the 4/10 form. More metabolites were up-regulated than down-regulated between all the time-points, but this phenomenon was most visible between the control and 11th day of drought period, with 34 metabolites increasing their abundance (**Figures 3C** and **4**). In the same period, only 21 metabolites increased their accumulation level in the form 7/6 (**Figures 3B** and **4**). Seventeen of these metabolites overlapped in both introgression forms (**Figure 4**). After rehydration, 44 metabolites presented a higher accumulation level, compared to the control conditions, in the form 4/10 (**Figure 3C**). Twenty-six of them belonged to the group of metabolites which significantly increased their abundance after 11 days of drought period and remained this elevated accumulation level or even increased it after rehydration. This trend was not observed in the 7/6 form, with only eight metabolites remaining the elevated abundance after rehydration (**Figure 4**). Moreover, 18 more metabolites, mainly carbohydrates, which were not accumulated under drought conditions, increased their abundance significantly after rehydration in the 4/10 form. This phenomenon was not observed in 7/6 form (**Figure 4**). These relations were also visible in the PCA, where the Principal Component 1 accounted for 47% of the variance, clearly separated the two forms with respect to the control values for the analyzed metabolites and indicated that during drought duration and rehydration the patterns of metabolite dynamics were different in both plants. For this separation, the highest contribution revealed not only the metabolites from the correlation groups number 4, 5, and 6 with most carbohydrates and both substrates and products of photosynthesis but also from the group number 3 with amino acids (**Figure 5** and **Supplementary Table S2**).

#### Amino Acids and Amines

The accumulation of amino acids from the correlation group number 3 (**Figure 5**) revealed a significant impact on genotypes and time-points separation according to the PCA Component 2 values (**Supplementary Table S2**). Although, a clear separation of genotypes was not in fact observed, it was simultaneously noticed that after 6 and 11 days of drought treatment in both introgression forms the accumulation level of most amino acids significantly increased, compared to control values (**Figures 2**, **4** and **5**). A higher accumulation level of aspartic acid, isoleucine, lysine, methionine, tryptophan and tyrosine in response to drought application in both introgression forms, and asparagine only in the form 7/6, was revealed. On the other hand, alanine, arginine, proline and valine increased their accumulation level after 11 days of drought only in the form 4/10 (**Figures 2** and **4**). These results demonstrated that the proline accumulation level unexpectedly decreased at the beginning of drought (the 3rd and the 6th day of drought) in both analyzed grass forms. However, when drought treatment progressed, proline abundance returned to the values observed in the control conditions in the form 7/6, and was even higher in the form 4/10 (**Figure 2**). The accumulation pattern of dimethylglycine-like amine was different between the two forms. In the form 4/10, its increased accumulation level after 11 days of drought without further change after rehydration, was revealed. Contrary, in the 7/6 form, a progressive decrease in dimethylglycine -like amine accumulation during drought period, compared to the control conditions, and a slight increase after rehydration to the level observed in the 4/10 form, was noticed (**Figure 2**).

#### Carbohydrates

With respect to the accumulation profiles of the identified carbohydrates, including substrates and products of photosynthesis, it was revealed that under the control conditions the form 7/6 had a higher accumulation level of F-1,6-2P, G-6-P 2 and glucose, compared to the form 4/10. The accumulation levels of sucrose and fructose did not differ significantly between the two forms, and only G-6-P 1 showed a higher abundance in the 4/10 form. However, after 11 days of drought the accumulation of F-1,6-2P and G-6-P 2 decreased, and G-6-P 1 and mannose-6-phosphate increased in the 7/6 form to the levels observed in the 4/10 form (**Figure 2**). For the main photosynthesis products: glucose, fructose, and sucrose a significant increase of accumulation level after 11 days of drought in the analyzed introgression forms was observed. No significant differences between the plants in fructose and sucrose accumulation levels were revealed, and only glucose showed a significantly higher abundance in the 7/6 form (**Figure 2**). After rehydration, in the 4/10 form all these compounds demonstrated higher accumulation levels, compared to the 7/6 form.

Other identified here carbohydrates presenting significant differences in an accumulation level between the analyzed introgression forms were *myo*-inositol and *myo*-inositol-1-phosphate. Their abundance increased in response to drought in both introgression forms, but was significantly higher in the form 4/10 (**Figure 2**). The accumulation level of gentiobiose increased after 11 days of drought period in both forms being higher in the 7/6 form. Melibiose and glycerol revealed a gradual decrease of their abundance during drought period in the form 7/6 and more or less stable level in the 4/10 (**Figure 2**).

#### Other Compounds

Sugar acids, such as galactaric acid, gluconic acid 2, glyceric acid, ribonic acid 2, ribonic acid like 1, saccharic acid and threonic acid revealed a significant increase of accumulation level after 11 days of drought in the 4/10 form, while in the form 7/6 rather a stable or even a decreased accumulation level in majority of sugar acids, was observed. Only galactaric acid showed an increased accumulation level during drought in this introgression form (**Figure 2**). The analyzed accumulation patterns for organic acids revealed differences between two forms. Five of six analyzed organic acids presented a higher accumulation level under drought treatment and rehydration in the form 4/10. In the form 7/6, caffeic acid, dehydroascorbic acid, and maleic acid showed a significant reduction in an accumulation level under drought. Only glutaric acid and shikimic acid accumulated significantly during drought treatment in this introgression form (**Figure 2**).

## Discussion

### Primary Metabolite Accumulation Profiles with Respect to Chloroplast Fructose Bisphosphate Aldolase Activity and Photosynthetic Capacity

Under mild water deficit conditions stomata closure is the main physiological factor responsible for inhibiting photosynthesis through a reduction of CO_2_ availability for the assimilation process (Lawlor, 2002). However, an important recent topic for discussion has become the mechanisms of non-stomatal limitations of photosynthesis associated also with metabolic factors (Flexas and Medrano, 2002), such as the Calvin cycle enzymes, among which pFBA accumulation level plays a crucial role (Haake et al., 1998, 1999; Uematsu et al., 2012). The pFBA is a key enzyme of the regeneration phase of this cycle, and its activity may be important for the regulation of photosynthesis intensity (Haake et al., 1998; Raines, 2003). It initiates the third regeneration phase of the Calvin cycle by catalyzing the reversible conversion of glyceraldehydes-3-phosphate and dihydroxyacetone phosphate to fructose-1,6-bisphosphate (F-1,6-2P), and condensation of sedoheptulose-1,7-bisphosphate from erythrose-4-phosphate and dihydroxyacetone phosphate (Raines and Lloyd, 2007). It has been proved in several studies that a changed accumulation level of pFBA in plants could influence the efficiency of photosynthesis (Haake et al., 1998, 1999; Uematsu et al., 2012). However, this relationship has not been confirmed for the forage grasses. Here, we try to fill this gap in our knowledge. Photosynthesis is the main source of substrates for carbohydrates, amino acids, and other cellular compounds production. These metabolites serve not only as a cell energy reservoir but often also as signaling molecules important for growth, development, and response to unfavorable conditions (Dolferus, 2014). Thus, an inhibition of photosynthesis usually has its reflection in growth, biomass production, and metabolite accumulation disturbances (Chaves et al., 2010). Perennial grasses accumulate large quantities of soluble carbohydrates in their leaves, simultaneously having a low amount of starch (Pollock and Cairns, 1991; Cairns, 2002). The observed higher accumulation level of most primary metabolites under control conditions and drought treatment, including glucose in the form 7/6, was probably due to a higher efficiency of photosynthesis in this form. This validates one of our hypotheses formulated in the introduction section. On the other hand, a higher accumulation level of particular primary metabolites, including all the analyzed substrates and products of photosynthesis after re-hydration accompanied a higher potential of recovery in the 4/10 form (Perlikowski et al., 2014).

Our results indicated that water deficit had a significant impact on the pFBA activity in the two analyzed introgression forms. However, after further re-watering that activity was closer to the control values (before stress application) in the 4/10 form, indicating its higher potential of metabolism recovery, after stress cessation. It was previously observed that the impaired expression of pFBA in transgenic lines of *Solanum tuberosum* (potato) affected plant growth, and negatively influenced carbohydrates accumulation, including phosphoryl substrates for photosynthesis and fructose, glucose, sucrose, and starch in ambient conditions. A lower level of pFBA accumulation also influenced the activity of other Calvin cycle enzymes, such as plastid fructose-1,6-bisphosphatase (Haake et al., 1998, 1999). However, the impact of decreased pFBA activity on photosynthesis rate and metabolite accumulation was observed only when less than 50% of pFBA activity was removed, but even under those conditions the accumulation levels of most important hexose phosphates were not impaired (Haake et al., 1998). In the present study, the impact of higher activity of pFBA on a higher photosynthesis efficiency, and a higher metabolite accumulation level in the 7/6 form, could be suggested, but in fact the difference between the analyzed plants for the activity of this enzyme was less than 25%. Also, the research performed on transgenic *Nicotiana tabacum* (tobacco) with overexpression of pFBA (Uematsu et al., 2012) clearly showed that an increased accumulation level of pFBA in plant tissues enhanced plant growth, biomass production, CO_2_ assimilation rate and ribulose-1,5-bisphosphate accumulation. Both analyzed here introgression forms reduced significantly their stomatal conductance during drought period, compared to the control conditions, and this process was associated with a reduction of CO_2_ assimilation levels in these two plants. However, as proved also in our earlier work, with closely similar levels of stomatal conductance during drought, the form 7/6 revealed simultaneously a significantly higher level of CO_2_ assimilation rate, compared to the form 4/10 (Perlikowski et al., 2014). In the current study, we demonstrated that this phenomenon was accompanied by higher accumulation and activity levels of pFBA in the form 7/6. Further research is required on the aldolase gene expression level using qRT-PCR as well as on the expression of genes coding other enzymes of the Calvin cycle. As far as we know, this report is the first one for the *Lolium-Festuca* forage grasses demonstrating crucial components of non-stomatal regulation mechanisms of CO_2_ assimilation level and photosynthetic capacity under drought conditions.

### Primary Metabolite Accumulation Profiles in Response to Water Deficit Conditions

As per our hypothesis, water deficit revealed a significant impact on the primary metabolism of *Lolium-Festuca* grasses. The differences in the observed alterations in metabolite profiles were well visible between the analyzed introgression forms, mainly with respect to osmoprotectant and signaling molecules.

#### Carbohydrates and Their Derivatives

Within a group of osmotic active compounds, soluble carbohydrates are especially important. Except their obvious central role at various levels of plant cell metabolism, these compounds are also associated with a wide range of response and signaling pathways altered by environmental stimuli (Hare et al., 1998; Fang and Xiong, 2015). Their small size, neutral character and biochemical compatibility allow them to maintain cellular water potential and interact with other cellular compound, stabilizing protein and membrane structures (Hoekstra et al., 2001; Xoconostle-Cazares et al., 2010). In the previous section, the accumulation profiles of the identified substrates and products of photosynthesis, were discussed. Here, in this paragraph, more potential functions of glucose, fructose, and sucrose in plant cell response to drought treatment, are considered. A higher accumulation level of glucose noticed in the introgression form 7/6 on the 11th day of drought period could also result in a higher ROS production in this form (Russell et al., 2002), and its higher exposure to potential oxidative damage, reducing the level of regeneration. However, this research aspect requires further work. An increased accumulation level of sucrose, fructose, and glucose was previously observed in forage grasses, such as *F. arundinacea* (Zwicke et al., 2015) and *L. perenne* (Foito et al., 2009), and in other plant species, such as *Oryza sativa* (rice; Ambavaram et al., 2014), *Triticum aestivum* (wheat; Izanloo et al., 2008), and *S. tuberosum* (Yang et al., 2015) under dehydration conditions, indicating that the mechanism of water deficit tolerance could be associated with a higher accumulation of soluble carbohydrates (Izanloo et al., 2008; Ambavaram et al., 2014). On the other hand, the research performed on *L. perenne* under drought treatment showed that sucrose, glucose, and fructose accumulation levels did not change significantly in leaves under stress treatment (Amiard et al., 2003). Glucose and sucrose are among the final products of carbon assimilation in plants, as well as precursors of the majority of organic compounds used in metabolic pathways (Ap Rees, 1995). Changing environmental conditions, including stress events such as water deficit often promote the accumulation of sucrose and products of its metabolism in the vegetative tissues. These compounds can serve as osmoprotectants (Kido et al., 2013), replacing dissipating water, especially under desiccation conditions (Hoekstra et al., 2001). The sucrose accumulation level is a result of sucrose biosynthesis or degradation of sugar polymers, such as starch. Both of these processes can be stimulated by drought (Zwicke et al., 2015). Overall, it was proved that sucrose could be associated with a dehydration avoidance mechanism, and can replace the functions of other osmoprotectants, such as trehalose or raffinose (Foito et al., 2009), which were not identified here.

Other well-recognized osmoprotectants revealed here involved gentiobiose, melibiose, and glycerol (Bartels and Sunkar, 2005), which presented genotype dependent differences in accumulation levels under drought conditions. The important metabolites identified here are also *myo*-inositol and its related compounds, which can be used as substrates for the synthesis other osmoprotectants, such as raffinose family oligosaccharides (Karner et al., 2004), galactinol (ElSayed et al., 2014) or d-ononitol (Sheveleva et al., 1997), and a wide spectrum of lipid signaling compounds, such as phosphatidylinositol, phosphatidylinositol-phosphate, polyphosphoinositides, *myo*-inositol phosphate or sphingolipid related molecules - crucial components of various metabolic pathways involved in a control of gene expression, hormonal regulation and response reactions to stress conditions (Liu et al., 2013; Zhai et al., 2015). *Myo*-inositol belongs to a sugar alcohol group of metabolites in which hydroxyl group can substitute the hydroxyl group of water during interaction with membrane lipids and proteins, maintaining their structure and properties (ElSayed et al., 2014). The increased accumulation of *myo*-inositol and *myo*-inositol-1-phosphate in the 4/10 form was positively correlated with a higher accumulation level of phosphatidylinositol in this form during drought treatment, as described earlier by Perlikowski et al. (2016). It was shown earlier that the overexpression of *myo*-inositol-1-phosphate synthase, which is a key enzyme restricting amount of produced *myo*-inositol, increased the level of tolerance to osmotic stresses in plants, such as rice, tobacco, and potato (Yang et al., 2008; Goswami et al., 2014; Zhai et al., 2015). Also, in the other experiments, in drought and salt treated tobacco *myo*-inositol was accumulated in higher amounts (Sheveleva et al., 1997). In this work, an increased abundance of *myo*-inositol did not perfectly reflect the accumulation level of its potential product – galactinol under drought conditions. On the other hand, the increase of *myo*-inositol and galactinol accumulation levels was not observed earlier in *L. perenne* under drought conditions (Amiard et al., 2003). The accumulated carbohydrates can above all serve as carbon storage for recovery period after stress cessation (Hare et al., 1998). A higher accumulation level of *myo*-inositol, *myo*-inositol-1-phosphate and melibiose after 11 days of drought in the form 4/10 could positively influence a faster recovery of this form during rehydration.

Shikimic acid is a key compound used in biosynthetic pathway of aromatic amino acid production, such as phenylalanine, tyrosine, and tryptophan (Kojima et al., 2015). Shikimic acid was previously observed to accumulate under drought in potato leaflets (Yang et al., 2015) and *L. perenne* leaves (Foito et al., 2009). An accumulation of known antioxidants, such as threonic acid and dehydroascorbic acid (Debolt et al., 2007) in the form 4/10 under drought and further recovery might be associated with more efficient regeneration mechanism in this form, associated with more efficient scavenging of ROS. In the form 7/6, those compounds were not accumulated highly during drought treatment, compared to the control conditions or even their accumulation levels decreased in drought.

#### Amino Acids

Amino acids represent a highly significant group of metabolites, and it was demonstrated that their accumulation levels have a significant impact on the expression of plant drought tolerance (Barchet et al., 2014). It was previously observed that a particular amino acid content increased under drought conditions in plants. In creosotebush (*Larrea divaricata*) a significant increase of alanine, arginine, histidine, isoleucine, valine, glutamic acid, phenylalanine, and proline abundance was observed (Saunier et al., 1968). Also potato plants exposed to drought treatment accumulated higher amounts of glutamine/glutamic acid, serine, threonine, proline, phenylalanine, isoleucine, leucine, and valine (Yang et al., 2015). The previously observed higher accumulation level of asparagine in *O. sativa* cultivars under drought conditions was found to be negatively correlated with drought tolerance, and was characteristic for susceptible genotypes with a lower water use efficiency (Degenkolbe et al., 2013). The amino acid accumulation in plants exposed to dehydration conditions could be due as well to a protein hydrolysis (Saunier et al., 1968), and may be also associated with nitrogen storage for further metabolism remobilization during recovery period after stress cessation (Sicher and Barnaby, 2012).

One of the most common reactions of many plants upon experiencing dehydration conditions, is an accumulation of proline and glycine betaine (Bandurska and Jóźwiak, 2010). Proline, with its neutral character, does not negatively affect the cell environment and could be accumulated in higher amounts, since it functions as an osmotic adjustment molecule and protects other cellular compounds, such as proteins and lipids against denaturation and peroxidation (Bandurska, 2001). Proline has a high water potential, and its hydrophobic site can interact with hydrophobic parts of other proteins, while a hydrophilic site with high affinity to water allows it to maintain a high water potential, solubility and a native structure of proteins under dehydration conditions (Hoekstra et al., 2001). It was previously reported that related genotypes of *F. arundinacea* (Abernethy and McManus, 1998), *F. rubra* and *L. perenne* (Bandurska and Jóźwiak, 2010; Salehi et al., 2014) accumulated higher levels of proline and glycine betaine during a water deficit period, compared to well-watered plants. On the other hand, it was proposed earlier that proline had a lower contribution to the osmotic adjustment under drought stress in forage grasses, compared to soluble carbohydrates (Barker et al., 1993). Glycine betaine accumulated in plants under water deficit conditions, functioning as an osmoprotector and stabilizing cell compounds structure and activity (Sakamoto and Murata, 2002). It was previously reported that a progressive drought treatment induced an accumulation of glycine betaine in *Hordeum vulgare* (barley; Zuniga et al., 1989) and *F. arundinacea* (Abernethy and McManus, 1998). Here, glycine betaine was not identified, although one of its relatives, a precursor dimethylglycine-like was found among the analyzed metabolites. Probably, a higher accumulation level of proline and dimethylglycine-like under the advanced drought conditions (the 11th day of drought) in the form 4/10 could positively affect a regeneration capacity of this form during recovery period, after rehydration.

### Primary Metabolite Accumulation Profiles and Plant Regeneration after Stress Cessation

A recovery after cessation of water deficit conditions refers to a plant’s ability for re-growth, and to produce a fresh biomass during tissue regeneration following severe turgor loss and dehydration (Luo, 2010). In previous work, it was shown that the form 4/10, after 10 days of rehydration, was characterized by a significantly decreased level of electrolyte leakage, compared to its level after 11 days of drought, whereas in the 7/6, an elevated level of electrolyte leakage was also observed after a rehydration period. This indicated that the form 4/10 had more efficient regeneration mechanisms after stress cessation (Perlikowski et al., 2014). During recovery, soluble compounds stabilizing membrane surface are replaced by water in rehydrating membranes allowing them to slowly regain their function, and preventing any quick rupture of membranes caused by water flow (Hoekstra et al., 2001). It has been noticed that the accumulation of osmotically active compounds during a drought period was not fully reversible after the stress cessation, and this was associated with a stress memory. The rate of possible return of these compounds to the control values depended mainly on the strength of earlier stress treatment and the level of plant stress tolerance (Morgan, 1984). Thus, we assume that observed elevated levels, after rehydration, of most crucial compounds of photosynthetic pathway and osmoprotectants in the 4/10 form could be, at least partially, associated also with a stress memory, and with a stronger physiological performance of this form under recovery conditions, compared to the form 7/6. Sucrose decreased its abundance in both introgression forms after rehydration, and this could be associated with metabolic demands to restore normal cell activity and growth after stress cessation (Zwicke et al., 2015). Osmotic adjustment is considered as one of the most important mechanisms of plant tolerance to water deficit conditions, and also plays a crucial role in a plant recovery after stress cessation (Morgan, 1984). The impact of drought stress on plant development depends mainly on the stress intensity and duration, but also on genotype specific traits and earlier plant pre-hardening in stressful conditions. Although all the mechanisms driving a stress memory are still poorly understood, the evidence exists that the accumulation of some signaling molecules during drought conditions could be, at least partially, associated with this phenomenon (Bruce et al., 2007).

## Conclusions

**FIGURE 6:**
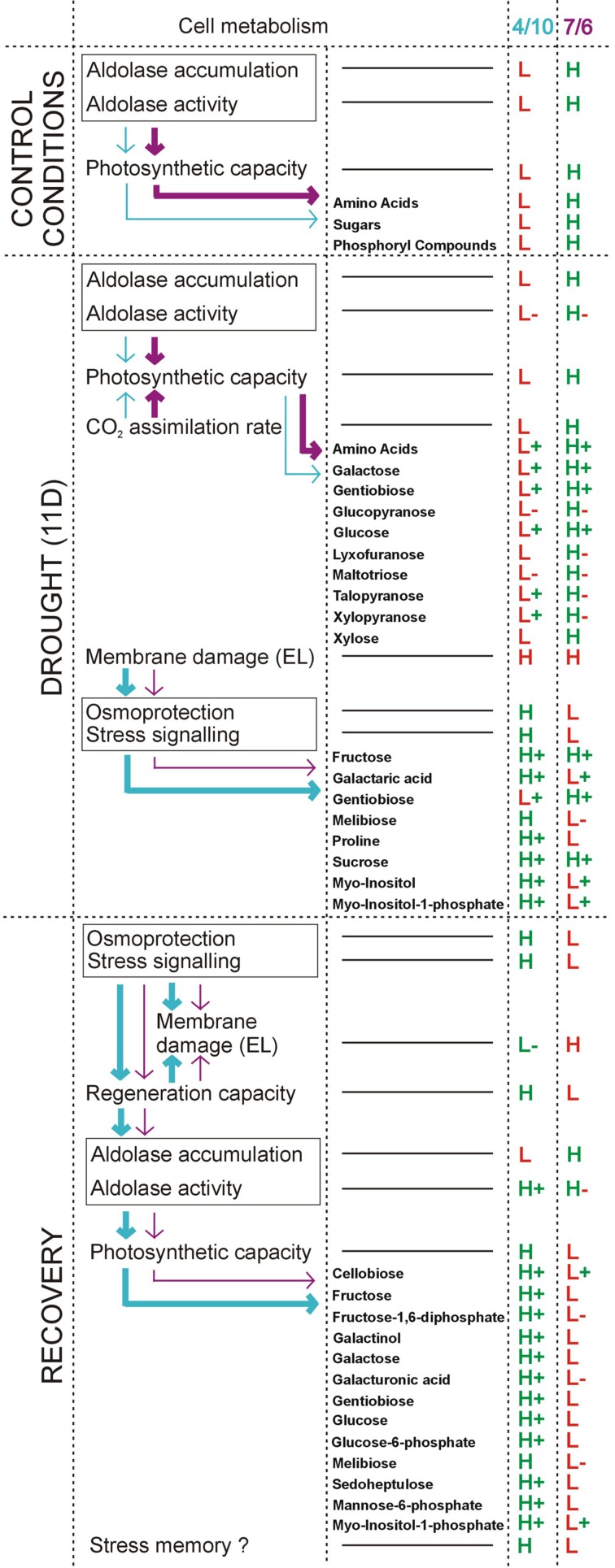
**A model figure showing the most important conclusions of the present study.** This model involves the results regarding chloroplast aldolase (pFBA) activity and specific metabolites’ accumulation levels under drought and recovery conditions in the 7/6 and 4/10 *L. multiflorum/F. arundinacea* introgression forms. In velvet – characteristics for the 7/6 form and in blue – for the 4/10 form, are presented. The intensity of the arrows shows quantitative differences in the indicated physiological/metabolic process between the analyzed introgression forms. Abbreviations: D, day; EL, electrolyte leakage; H, higher level, compared to the other introgression form; L, lower level, compared to the other introgression form; (+), increased level, compared to the control conditions; (-), decreased level, compared to the control conditions.

The results obtained in this study clearly indicate that accumulation and activity levels of pFBA can influence the capacity of photosynthesis in the *L. multiflorum/F. arundinacea* introgression forms, due to the efficiency of the Calvin cycle. The phenomenon, described in our paper, is the first example of non-stomatal mechanisms involved in a regulation of photosynthetic rate during prolonged drought treatment in the *Lolium-Festuca* forage grasses. The higher activity and accumulation levels of pFBA in the 7/6 form was accompanied by a higher accumulation level of most photosynthesis products in this form, during control and drought periods. On the other hand, the form 4/10 demonstrated higher accumulation levels of stress tolerance marker metabolites, such as proline, melibiose, galactaric acid, *myo*-inositol and *myo*-inositol-1-phosphate under drought conditions. Their accumulation could be associated with more efficient capacity of membrane regeneration in this form, after stress cessation. Furthermore, during rehydration, the 4/10 introgression form also maintained elevated levels of most metabolites, analyzed herein. It cannot be excluded though that these alterations to metabolism could be involved in a stress memory mechanism in forage grasses, however, this research aspect requires further work. The most important conclusions of this study are presented also graphically in a model figure (**Figure 6**).

## Author Contributions

DP and AK designed the experiments. DP, MC, ŁM, and AA conducted the experimental work. DP and AK drew main conclusions. DP carried out the statistical analysis. DP and AK prepared the first version of the manuscript, but all the authors contributed in further writing, and finally read and approved the manuscript.

## Conflict of Interest Statement

The authors declare that the research was conducted in the absence of any commercial or financial relationships that could be construed as a potential conflict of interest.

## Footnotes

1 www.agrisera.com

2 http://gmd.mpimp-golm.mpg.de

## Supplementary Material

**FIGURE S1 | The alignment of chloroplast fructose bisphosphate aldolase (pFBA) cDNA sequences with 3′ and 5′ UTR of 4/10 and 7/6 *Lolium multiflorum/Festuca arundinacea* introgression forms with pFBA cDNA sequence of *Brachypodium distachyon* (B.d.; XM_003577737.1).** Gray highlighted text indicates ‘start’ and ‘stop’ positions of the coding sequence.

**FIGURE S2 | The alignment of chloroplast fructose bisphosphate aldolase (pFBA) predicted protein sequences of 4/10 and 7/6 *L. multiflorum/F. arundinacea* introgression forms with pFBA protein sequence of *Brachypodium distachyon* (B.d.; XM_003577737.1).** Black highlighted text indicates a predicted chloroplast transition sequence.

**TABLE S1 | The normalized mass spectral intensities for three biological replicates, data base annotations and ANOVA results of the analyzed metabolites.** Calculations were performed for five time-points of experiment: before drought, after three, six and 11 days of drought, and after ten days of re-watering in *Lolium multiflorum/Festuca arundinacea* introgression forms, based on [Log_2_] transformed normalized mass spectra peak intensities.

**TABLE S2 | Loadings of all the PCA components for the analyzed metabolites.** Calculations were performed for five time-points of experiment: before drought, after three, six and 11 days of drought, and after ten days of re-watering in *Lolium multiflorum/Festuca arundinacea* introgression forms, based on [Log_2_] transformed normalized mass spectra peak intensities.

## Abbreviations

F-1,6-2P: Fructose-1,6-diphosphate
GC: gas chromatography
G-6-P: Glucose-6-phosphate
MS: mass spectrometry
MSTFA: *N*-Methyl-*N*-(trimethylsilyl) trifluoroacetamide
PCA: principal component analysis
pFBA: plastid Fructose-1,6-bisphosphate aldolase
RACE: rapid amplification of cDNA ends
ROS: reactive oxygen species
UTR: untranscribed region
