# Supplementary material for: Water Deficit Affects Primary Metabolism Differently in Two Lolium multiflorum/Festuca arundinacea Introgression Forms with a Distinct Capacity for Photosynthesis and Membrane Regeneration
Source: Front Plant Sci. 2016 Jul 25;7:1063. doi: 10.3389/fpls.2016.01063 (PMC4958636; doi:10.3389/fpls.2016.01063)
Supplement: Supplementary file 2 [file Image_2.PDF]

```
                    10        20        30        40        50        60        70        80        90       100
            ....|....|....|....|....|....|....|....|....|....|....|....|....|....|....|....|....|....|....|....|
pFBA-1 7/6  MASATLLKSSFLPKKSEWGVTRQAAAPKPMTVSMVVRASAYADELIKTAKTIASPGRGILAMDESNATCGKRLASIGLENTEANRQAYRTLLVTPPGLGN
pFBA-2 7/6  ........................V...........................................................................
pFBA-1 4/10 ....................................................................................................
pFBA-2 4/10 ........................V...........................................................................
pFBA B.d.   .....I.............A.....T..Q................V...N.......................D..........................

                   110       120       130       140       150       160       170       180       190       200
            ....|....|....|....|....|....|....|....|....|....|....|....|....|....|....|....|....|....|....|....|
pFBA-1 7/6  YISGAILFEETLYQSTVDGKKIVDILVEQGIVPGIKVDKGLVPLVGSNDESWCQGLDGLASREAAYYQQGARFAKWRTVVSIPNGPSELAVKEAAWGLAR
pFBA-2 7/6  ....................................................................................................
pFBA-1 4/10 ....................................................................................................
pFBA-2 4/10 ....................................................................................................
pFBA B.d.   ....................................................................................................

                   210       220       230       240       250       260       270       280       290       300
            ....|....|....|....|....|....|....|....|....|....|....|....|....|....|....|....|....|....|....|....|
pFBA-1 7/6  YAAISQDNGLVPIVEPEIMLDGEHGIERTFEVAQKVWAETFYYMAQNNVMFEGILLKPSMVTPGAECKDRNTPEEVASYTLKLLQRRIPPSVPGIMFLSG
pFBA-2 7/6  ....................................................................................................
pFBA-1 4/10 ..............G.....................................................................................
pFBA-2 4/10 ......................................................................................K.............
pFBA B.d.   ..................L.......D......................L..................E.A...Q.........................

                   310       320       330       340       350       360       370       380
            ....|....|....|....|....|....|....|....|....|....|....|....|....|....|....|....|....|...
pFBA-1 7/6  GQSEVEATLNLNAMNQSPNPWHVSFSYARALQNTCLKTWGGRPENVAAAQEALLLRAKANSLAQLGKYTSDGEAAAAKEGMFVKNYSY
pFBA-2 7/6  ........................................................................................
pFBA-1 4/10 ........................................................................................
pFBA-2 4/10 ........................................................................................
pFBA B.d.   ........................................................................................
```
